# Chronic Complications Following Dengue Infection: A Scoping Review

**DOI:** 10.7759/cureus.116633

**Published:** 2026-09-21

**Authors:** Franklin Joel Viera Heredia, Emilio José Zambrano Narváez, Anahí Milena Yánez Pilco, Thalia Mishell Escudero Andino, Daniela Alejandra Fernández Gómez, Nicolás Andre Gallardo Larreina, Paola Alejandra Fuentes Lara, Laura Azalea Salazar Heredia

**Affiliations:** 1 Medicine and Surgery, Pontificia Universidad Católica del Ecuador, Quito, ECU; 2 General Practice, Ministerio de Salud Pública, Quito, ECU; 3 Surgery, Ministerio de Salud Pública, Quito, ECU; 4 General Medicine, Ministerio de Salud Pública, Quito, ECU; 5 Medicine, Universidad Nacional de La Plata, La Plata, ARG

**Keywords:** chronic sequelae, dengue infection, latin america, neurological complications, ophthalmological complications, post-dengue syndrome, psychiatric complications, scoping review, viral neurotropism

## Abstract

Dengue virus infection has increased substantially in Latin America, prompting epidemiological alerts from regional health authorities in recent years. While most published research focuses on the acute phase, chronic multisystemic sequelae remain poorly characterized. This scoping review aimed to identify and describe chronic complications in adult patients following dengue infection, as reported in the available scientific literature.

A scoping review was conducted following the PRISMA-ScR guidelines. A structured Population, Concept, Context (PCC) question guided searches of PubMed (National Library of Medicine, Bethesda, Maryland), Scopus (Elsevier, Amsterdam, the Netherlands), Google Scholar (Google, Mountain View, California), EMBASE (Elsevier, Amsterdam, the Netherlands), Cochrane Library (John Wiley & Sons, Hoboken, New Jersey), and LILACS (BIREME, São Paulo, Brazil), and gray literature from January 2005 to June 15, 2026. Articles were screened independently by eight reviewers using Rayyan; inter-rater reliability was assessed using Fleiss' kappa (κ = 0.81, 95% CI: 0.74-0.88), and disagreements were resolved by consensus. Of the 222 identified records, 62 studies met the inclusion criteria after deduplication and full-text screening. Neurological complications were the most frequently represented category among the included publications (30.6%, n = 19), followed by ophthalmological (19.4%, n = 12), heterogeneous persistent symptoms (12.9%, n = 8), fatigue (9.7%, n = 6), multisystemic (9.7%, n = 6), psychiatric (9.7%, n = 6), cardiological (4.8%, n = 3), and immunological (3.2%, n = 2) complications. These proportions reflect the distribution of the published literature rather than the clinical prevalence of complications among dengue patients, given that approximately one-third of the included studies were case reports or case series. The evidence base includes the largest population-based cohort of post-acute dengue sequelae to date (n = 55,870, Singapore), a two-year disability-adjusted life-year (DALY) burden analysis from the same registry, the first systematic review of dengue-associated Guillain-Barré syndrome, the first reported case of chronic dengue encephalitis with brain-tissue DENV RNA confirmed by metagenomic sequencing, and two narrative reviews that substantially expanded the evidence base on psychiatric sequelae.

Dengue infection is associated with a broad and heterogeneous spectrum of chronic complications affecting multiple organ systems, including elevated post-acute cardiovascular, neuropsychiatric, and autoimmune risks, as well as a measurable disease and healthcare cost burden. The absence of a consistent relationship between acute severity and chronic outcomes supports structured, evidence-based, long-term post-dengue follow-up, particularly for patients with persistent symptoms or clinically relevant risk factors and for underrepresented Latin American populations.

## Introduction and background

Dengue virus (DENV) is a positive-sense single-stranded RNA flavivirus transmitted primarily by *Aedes aegypti* and, to a lesser extent, *Aedes albopictus* mosquitoes. It comprises four antigenically distinct serotypes (DENV-1 through DENV-4), each capable of causing clinically indistinguishable illness. However, infection with one serotype confers only serotype-specific long-term immunity. The World Health Organization estimates that approximately 100-400 million dengue infections occur annually, with roughly 40% of the world's population currently at risk; the number of notified cases increased tenfold between 2000 and 2019, from approximately 500,000 to over 5.2 million across more than 80 countries [1].

Clinically, dengue infection is classified into three sequential phases: the febrile phase (days 1-7), characterized by sudden-onset high fever and myalgia; the critical phase (days 3-7), defined by plasma leakage and thrombocytopenia; and the recovery phase (days 7-10), during which reabsorption of extravasated fluids occurs. Disease severity spans a spectrum from dengue without warning signs to dengue with warning signs and severe dengue, the latter encompassing dengue hemorrhagic fever and dengue shock syndrome [1]. This well-characterized acute presentation has shaped the dominant clinical and research focus on short-term management and the reduction of case fatality. Despite the extensive literature on acute dengue, the chronic multisystemic sequelae that emerge or persist beyond the recovery phase remain comparatively understudied [2]. No universally accepted definition or classification framework exists for subacute or chronic complications following dengue infection. For this review, chronic complications were operationally defined as clinical manifestations, symptoms, or sequelae that persisted or emerged more than four weeks after the onset of dengue symptoms. The four-week threshold was selected because it substantially exceeds the typical duration of the acute and recovery phases of dengue infection (up to approximately 10 days), providing a conservative, clinically distinguishable cutoff between expected acute-phase resolution and true chronic persistence, consistent with thresholds used elsewhere in the post-infectious and post-viral syndrome literature. This threshold was selected to distinguish persistent or late manifestations from complications confined to the expected acute and early recovery phases. Because certain neurological and cardiovascular conditions, including Guillain-Barré syndrome (GBS) and cardiomyopathies, may begin during the acute or early post-acute period but result in persistent disability or long-term sequelae, these conditions were eligible when persistent manifestations beyond the four-week threshold were documented [3,4]. This operational definition therefore encompasses two distinct but overlapping constructs: (i) post-acute persistent or new-onset symptoms with a delayed or gradual course (e.g., post-dengue fatigue, persistent arthralgia), and (ii) long-term sequelae resulting from irreversible tissue damage sustained during the acute phase (e.g., residual neurological deficits after stroke or peripheral nerve injury) when disability persisted beyond four weeks. Both categories were included under the umbrella of "chronic complications" for the purposes of this review.

The pathophysiological mechanisms proposed to contribute to post-dengue chronicity are multifactorial, involving direct viral neurotropism, immune-mediated mechanisms such as molecular mimicry and autoantibody production, persistent cytokine dysregulation, hypothalamic-pituitary-adrenal (HPA) axis disruption, and autonomic nervous system dysregulation [5,6]. However, the relative contribution of each mechanism to specific chronic complications and the extent to which these pathways are supported by direct clinical or laboratory evidence remain unclear. This scoping review aims to systematically map and describe the chronic complications reported following dengue infection in adults across organ systems, characterize their underlying evidence base and proposed pathophysiological mechanisms, and identify gaps in the literature (particularly the scarcity of prospective, population-level, and Latin American data) to inform future research and post-dengue clinical follow-up.

## Review

Methods

Study Design

We conducted a scoping review following the Preferred Reporting Items for Systematic Reviews and Meta-Analyses extension for Scoping Reviews (PRISMA-ScR) framework. The guiding research question was, what chronic complications have been reported following dengue infection in adult patients, and what is their frequency, onset, duration, and underlying evidence base across organ systems and geographic regions? This question was structured using the PCC framework: Population (adult patients with prior dengue infection confirmed by serology or PCR), Concept (chronic complications across cardiovascular, immunological, neurological, ophthalmological, psychiatric, and multisystemic domains), and Context (adult populations recovering from dengue infection worldwide, as reported in the international scientific literature). The review protocol was registered with the Open Science Framework (OSF; osf.io/7dvuj, Center for Open Science, Charlottesville, Virginia) on May 30, 2026. Database searches had commenced before registration; therefore, the registration was retrospective relative to the initiation of the search process. This timing is reported transparently as a methodological limitation.

Search Strategy

A structured search covering January 2005 to June 15, 2026, was conducted across PubMed (National Library of Medicine, Bethesda, Maryland), Google Scholar (Google, Mountain View, California), Scopus (Elsevier, Amsterdam, the Netherlands), EMBASE (Elsevier, Amsterdam, the Netherlands), Cochrane Library (John Wiley & Sons, Hoboken, New Jersey), LILACS (BIREME, São Paulo, Brazil), and gray literature sources. Gray literature was manually searched through institutional repositories and websites of tropical medicine societies and universities in Brazil, Colombia, Ecuador, Mexico, and Peru to capture regionally relevant Latin American findings not indexed in the major databases above (complete search strategies by database, including gray literature, are detailed in Appendices).

The search terms included "dengue," "dengue fever," "post-infection," "chronic," "long term," "complications," "sequelae," and "acute," combined using the Boolean operators AND, OR, and NOT. Filters were applied for language and study population (human adults). Full-text articles were retrieved from open-access repositories and through institutional subscriptions. Complete search strings and database yields are provided in Appendices.

Eligibility Criteria

Inclusion criteria: (1) adult population (≥18 years); (2) serologically or PCR-confirmed dengue diagnosis; (3) chronic complications, defined as those appearing after four weeks post-infection or following a natural course exceeding one month (with the exceptions noted above for GBS and cardiomyopathies). Exclusion criteria: (1) articles published before 2005; (2) exclusively pediatric populations; (3) descriptions limited to acute-phase complications resolving within four weeks; (4) absence of serological/PCR confirmation; (5) and inaccessible full text.

Study Selection and Inter-rater Reliability

Identified records were imported into Rayyan for duplicate removal and eligibility screening. Eight independent reviewers screened titles and abstracts, followed by full-text assessment of potentially eligible articles. Blind mode in Rayyan was strictly maintained during title- and abstract-level screening by all eight reviewers to prevent selection bias and was unblinded only after initial decisions were logged for consensus resolution. Inter-rater reliability was formally assessed using Fleiss' kappa coefficient, which was appropriate given the involvement of eight independent reviewers in the screening. The kappa value obtained was κ = 0.81 (95% CI: 0.74-0.88), indicating strong agreement among reviewers. Disagreements were resolved by consensus.

Data Extraction and Quality Assessment

Data extraction and Joanna Briggs Institute (JBI) quality appraisal were performed independently by pairs of reviewers (four pairs drawn from the same eight-reviewer screening team), each assigned a subset of the 62 included studies; disagreements were first resolved through discussion within each pair and, when unresolved, adjudicated by the senior/supervising author.

A standardized extraction matrix was used to record the authors, publication year, country, study design, study objectives, DOI/link, and primary complication category. For the purposes of this review, an included "study" was defined as an eligible publication or report that met the predefined inclusion criteria, regardless of study design. Each included study was assigned a primary complication category based on the principal chronic outcome investigated. When a study reported outcomes involving multiple organ systems, it was assigned to the category corresponding to its primary outcome or predominant complication domain. Studies addressing multiple organ systems without a predominant domain were classified as multisystemic.

The methodological quality of all 62 included studies was assessed using the JBI critical appraisal checklists appropriate to each study design and is summarized in Table 1. Design-specific JBI checklists were applied (cohort studies for cohorts; systematic reviews for systematic reviews; case series; case reports; analytical cross-sectional studies for cross-sectional designs; and text and opinion papers for narrative reviews), with quality scored as the percentage of "yes" items met and categorized a priori as high (≥75%), moderate (50-74%), or low (<50%), per conventional JBI thresholds. As each checklist evaluates design-specific domains, these categories reflect within-design rigor rather than a directly comparable cross-design scale. No meta-analysis or pooled statistical synthesis was planned or performed, given the substantial heterogeneity in study designs, populations, outcome definitions, and follow-up durations across included studies; findings are instead synthesized narratively, with counts, percentages, and effect estimates (e.g., relative risks, hazard ratios, and 95% CIs) reported as originally presented by the primary studies.

**Table 1:** Joanna Briggs Institute (JBI) methodological quality assessment by study design, all 62 included studies. Quality categories: high (≥75%), moderate (50-74%), and low (<50%) of criteria met.

Study design	n studies	High quality (≥75%)	Moderate (50-74%)	Low (<50%)
Prospective cohort	11	9 (82%)	2 (18%)	0
Retrospective cohort	10	9 (90%)	1 (10%)	0
Systematic review	4	4 (100%)	0	0
Case series	3	1 (33%)	2 (67%)	0
Case report	17	8 (47%)	6 (35%)	3 (18%)
Other (cross-sectional/narrative)	17	4 (24%)	10 (59%)	3 (18%)
Total	62	35 (56%)	21 (34%)	6 (10%)

Results

Study Selection

The search identified 222 raw records; 52 duplicates were removed, leaving 170 unique records for title/abstract screening. Seventeen were excluded at this stage, and 153 reports were sought and assessed at full text. Ninety-one were excluded (53 acute-phase-only, 15 with inaccessible or restricted full text, 12 addressing unrelated topics, and 11 exclusively pediatric), yielding 62 studies included in the final synthesis (Figure 1).

**Figure 1:**
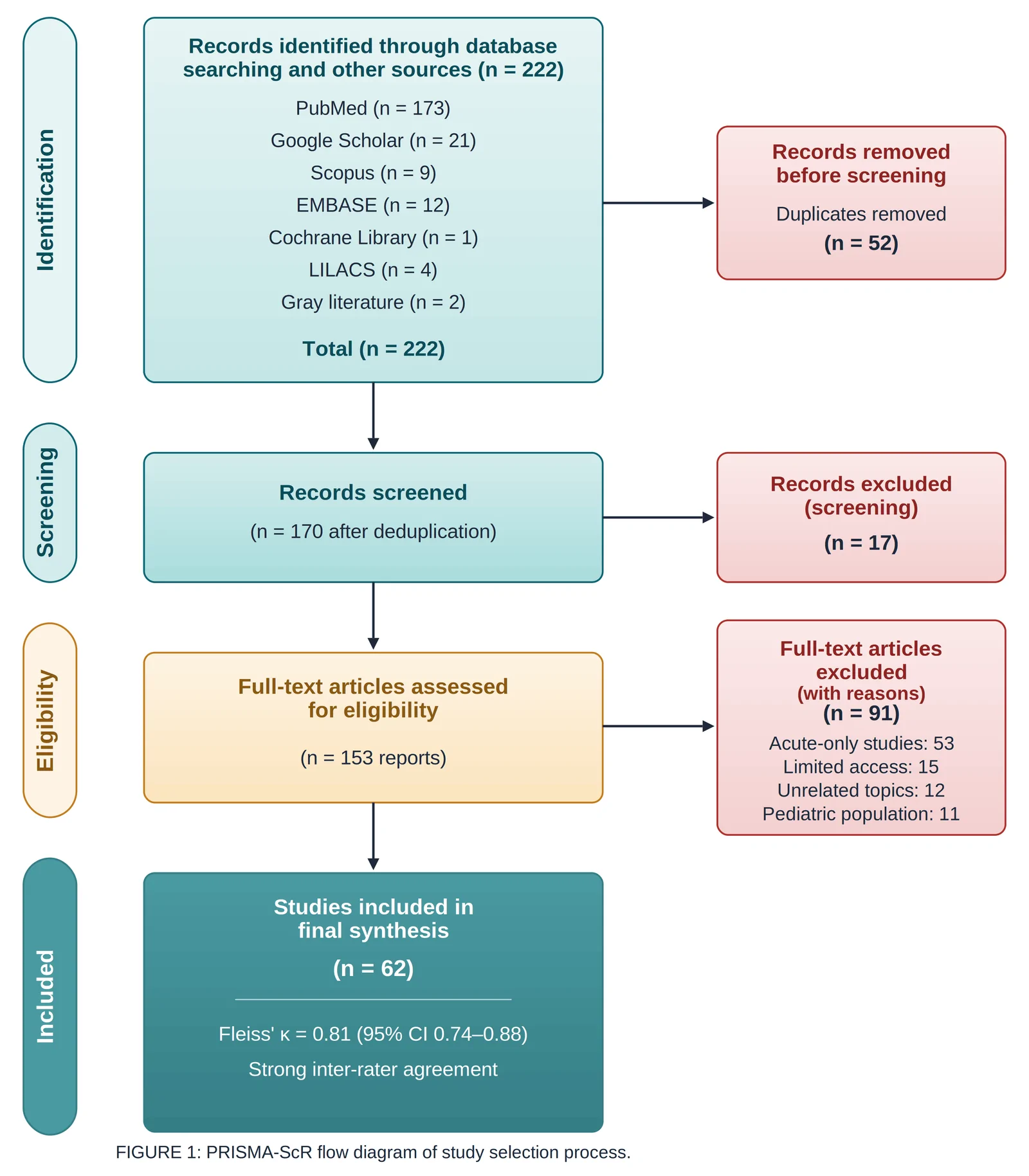
PRISMA-ScR flow diagram of study selection process. CI: confidence interval; κ: kappa statistic; LILACS: Latin American and Caribbean Health Sciences Literature; PRISMA-ScR: Preferred Reporting Items for Systematic Reviews and Meta-Analyses extension for Scoping Reviews.

Characteristics of Included Studies

Of the 62 included studies, the majority were indexed in PubMed, with additional records identified via Google Scholar, Scopus, and EMBASE. Study designs varied, comprising prospective and retrospective cohort studies, including several large population-based cohorts from Singapore, Sudan, and Brazil, systematic and narrative reviews, case series, and case reports. Methodological quality also varied across study designs: 35 studies (56%) were classified as high quality, 21 (34%) as moderate quality, and 6 (10%) as low quality according to the predefined JBI criteria (Table 1). Notably, case reports and case series constituted an important proportion of the evidence base and were interpreted primarily as signals of rare or clinically distinctive manifestations rather than as evidence of population-level frequency or causal association. The countries contributing the most studies were Singapore (n = 10), followed by India (n = 8), Sri Lanka (n = 7), and Brazil and Taiwan (n = 6 each). Latin American countries (Brazil, Mexico, Colombia, Cuba, Ecuador, and Guatemala) contributed a combined 17 of 62 studies (27%), underscoring a persistent regional evidence gap relative to the region's dengue burden. Publications on chronic post-dengue sequelae have increased markedly in recent years, with 2025-2026 contributing the largest population-based cohort study on this topic to date [7] and the first population-level two-year disability-adjusted life-year (DALY) burden estimate [8].

Distribution by Complication Type

Across the 62 included studies, neurological complications were the most frequently represented (30.6%, n = 19), followed by ophthalmological (19.4%, n = 12), heterogeneous persistent symptoms (12.9%, n = 8), fatigue-related (9.7%, n = 6), multisystemic (9.7%, n = 6), psychiatric (9.7%, n = 6), cardiological (4.8%, n = 3), and immunological (3.2%, n = 2). These percentages reflect the proportion of included publications addressing each complication category and should not be interpreted as estimates of clinical prevalence, incidence, or relative risk among dengue patients; approximately one-third of the included studies were case reports or case series, which are not designed to estimate population-level frequency. The multisystemic and psychiatric categories are substantially strengthened by recent large-cohort and review-level evidence, discussed in the corresponding sections below.

Complications by system

Cardiovascular Complications

Three studies addressed chronic cardiovascular sequelae following dengue infection (Table 2), converging on structural and functional myocardial involvement without a consistent relationship to dengue severity: proposed mechanisms include hydroelectrolytic imbalance, hypoperfusion, and lactic acidosis, leading to right ventricular dilatation, tricuspid regurgitation, and arrhythmias, alongside detectable echocardiographic myocardial alterations with a male predominance [4,9]; a broader multi-pathogen review of post-infectious sequelae, in which dengue was discussed among several arboviruses, corroborated susceptibility to similar cardiac sequelae [2]. These mechanistic findings are consistent with population-level data from the Singapore national registry, which reported a 46% higher post-acute cardiovascular risk (see Multisystemic Complications section below) [7]; because this estimate derives from the same registry discussed throughout the Multisystemic section, it should be regarded as hypothesis-strengthening rather than independent external confirmation.

**Table 2:** Summary of studies on cardiovascular complications.

Authors	Country/design	Main finding
Izquierdo-Condoy et al. [2], 2024	Ecuador - narrative review	Arboviruses can cause arrhythmias, myocarditis, pericarditis, pericardial effusion, and myocardial injury with chronic sequelae.
Kaagaard et al. [9], 2022	Brazil - cross-sectional cohort	Dengue infection induces structural and functional myocardial alterations, mainly in the left ventricle in males.
Shivanthan et al. [4], 2015	Sri Lanka - systematic review	Cardiac involvement is not uncommon and may be associated with significant morbidity.

Post-Dengue Fatigue

Six studies examined fatigue as a chronic post-dengue complication (Table 3). Among the primary cohort studies, reported prevalence ranged widely (17.3-81.5%) depending on follow-up duration and case definition: Perera et al. [10] reported fatigue in 81.5% of patients at hospital discharge and 35% at one month, and Sigera et al. [11] documented 32% persistence at two months (RR 4.93 vs. non-dengue). Garg [12], a narrative review rather than a primary study with an independent patient cohort, reported a summary estimate of 24.4% at two months that is not directly comparable to the primary-study figures above, while Umakanth [13] reported 17.3% at longer follow-up. Female sex, advanced age, chills, and absence of rash were identified as risk factors [14,15], whereas acute severity did not consistently correlate with its persistence [15,16].

**Table 3:** Summary of studies on post-dengue fatigue.

Authors	Country/design	Main finding
Garg [12], 2024	India - narrative review	24.4% of patients had fatigue two months after acute infection.
Perera et al. [10], 2023	Sri Lanka - descriptive longitudinal cohort	Fatigue present in 81.5% at discharge; 35% at one month.
Sigera et al. [11], 2021	Sri Lanka - prospective cohort	Fatigue in 32% at two months (RR 4.93 vs. non-dengue; RR 2.45 in women).
Umakanth [13], 2018	Sri Lanka - prospective descriptive	17.3% post-dengue fatigue syndrome with muscle weakness, pain, and poor concentration.
Luengas et al. [16], 2015	Colombia - cross-sectional descriptive	Fatigue prevalence of 34.4%.
Seet et al. [14], 2007	Singapore - prospective cohort	Risk factors: female sex, chills, advanced age, and absence of rash.

Heterogeneous Persistent Symptoms

Eight studies described persistent symptoms not attributable to a single organ system (Table 4), most consistently fatigue, arthralgia, myalgia, and headache. Among the primary cohort studies, González et al. [17] described symptoms persisting in 50% of patients at six months, and Schulte et al. [18] documented functional impairment for up to six months. Tiga et al. [19], in a narrative review, additionally reported high annual costs and functional limitations extending up to two years, associated with age and female sex. Teixeira et al. [20] identified symptoms in 54% of patients at 14 days, decreasing to 6.2% beyond 10.6 months, and Zeng et al. [21], in a narrative review, reported a summary prevalence of approximately 34%. Tristão-Sá et al. [22] documented persistent symptoms in 33% of patients at two months, with elevated transaminases in some cases, and in a separate cohort, Teixeira et al. [23] found that weakness, hyporexia, and somnolence predominated, with higher risk among women (OR 5.4). As narrative reviews without an independent patient cohort, the Tiga and Zeng estimates are presented here as supporting context rather than directly comparable prevalence figures. Risk factors included advanced age, female sex, and DENV-3 serotype.

**Table 4:** Summary of studies on heterogeneous persistent symptoms. DALY: disability-adjusted life-year.

Authors	Country/design	Main finding
Schulte et al. [18], 2020	Mexico - prospective cohort	Reduced health-related quality of life for up to six months.
Zeng et al. [21], 2018	USA - narrative review	~34% persistent symptoms, mean duration of one month.
Teixeira et al. [20], 2017	Brazil - prospective cohort	Symptoms in 54% at 14 days and 6.2% at ≥10.6 months.
Tiga et al. [19], 2016	Mexico - narrative review	High annual costs and DALY loss; associated with age and female sex.
Tristão-Sá et al. [22], 2012	Brazil - prospective cohort	Persistent symptoms in 33% at two months; elevated transaminases in some cases.
García et al. [15], 2011	Cuba - prospective cohort	>50% reported persistent symptoms over two years.
Teixeira et al. [23], 2010	Brazil - prospective cohort	Weakness, hyporexia, and somnolence predominant; higher in women (OR 5.4).
González et al. [17], 2005	Cuba - longitudinal cohort	50% with heterogeneous symptoms at six months.

Immunological Complications

Two studies addressed chronic immunological complications following dengue infection (Table 5). Shih et al. [24] reported a short-term increased risk of autoimmune encephalomyelitis mediated by cross-reactive antibodies against endothelial cells, platelets, and plasminogen. Rao et al. [25], in a broader narrative review of proximal myopathy etiologies, listed dengue virus among several viral infections (including HIV, influenza, and hepatitis B/C) associated with muscle weakness, without specifically characterizing a dengue-associated necrotizing myopathy subtype. Population-level data from Lim et al. [7], drawn from the same Singapore national registry cohort discussed throughout the Multisystemic Complications section, show a 37% elevated risk of autoimmune sequelae at 31-300 days post-infection (aHR 1.37, 95% CI 1.24-1.52) in a cohort of 55,870 adults, providing hypothesis-strengthening quantitative support for the autoimmune hypothesis raised by case-level studies, pending independent validation in other populations. This finding strengthens the call for dedicated prospective autoimmune-screening cohorts (ANA, anti-dsDNA, antiphospholipid antibodies, and muscle-specific kinase antibodies) in post-dengue patients, particularly in Latin American and South Asian settings currently underrepresented in this evidence base.

**Table 5:** Summary of studies on immunological complications.

Authors	Country/design	Main finding
Shih et al. [24], 2023	Taiwan - population-based cohort	Short-term increased risk of autoimmune encephalomyelitis post-dengue.
Rao et al. [25], 2022	Pakistan - narrative review	Dengue virus listed among viral causes of proximal muscle weakness (broader myopathy review).

Multisystemic Complications

Six studies addressed multisystemic complications (Table 6), including four large Singaporean population-based cohorts and complementary case-level evidence from other regions, which together provide the most robust quantitative evidence in this review. Also included is a narrative review with broader neurological and ophthalmological scope [5], retained here for its cross-system perspective on post-dengue chronicity. Wee et al. [26] reported a 21% higher overall sequelae risk and a 55% higher cardiovascular risk at 30-300 days. Lin et al. [6] found an increased risk of GBS, Parkinson's disease, dementia, and psychiatric disorders.

**Table 6:** Summary of studies on multisystemic complications following dengue infection. DALY: disability-adjusted life-year.

Authors	Country/design	Main finding
Guzmán and Martínez [5], 2024	Cuba - narrative review	Neurological symptoms may persist ≥6 months; ocular complications can cause irreversible disability.
Wee LE et al. [26], 2024	Singapore - retrospective cohort	21% higher overall sequelae risk; 55% higher cardiovascular risk at 30-300 days.
Lin HC et al. [6], 2024	Taiwan - retrospective cohort	Increased risk of GBS, Parkinson's disease, dementia, and psychiatric disorders.
Lim JT et al. [7], 2025	Singapore - population-based retrospective cohort (n = 55,870)	+19% any sequelae; +46% cardiovascular; +29% neuropsychiatric; +37% autoimmune at 31-300 days.
Chow JY et al. [8], 2026	Singapore - retrospective cohort (two-year)	First quantification of two-year DALY burden; elevated hospitalization risk persisting 24 months.
Lim JT et al. [27], 2025	Singapore - population-based cohort (costs)	11.9× higher acute inpatient rates; USD 687,032 excess post-acute healthcare costs.

Lim et al. [7] conducted the largest population-based retrospective cohort study of post-acute dengue sequelae to date, including 55,870 laboratory-confirmed dengue-infected adults and 3,072,309 uninfected controls (Singapore national registries, January 2017 to June 2023). At 31-300 days post-infection, dengue-infected individuals had a 19% higher risk of any sequelae (aHR 1.19, 95% CI: 1.13-1.26), a 46% higher cardiovascular risk (aHR 1.46, 95% CI: 1.01-2.10), with the confidence interval approaching the null and indicating a comparatively less precise estimate than those for the other domains reported, a 29% higher neuropsychiatric risk (aHR 1.29, 95% CI: 1.14-1.45), and a 37% higher autoimmune risk (aHR 1.37, 95% CI: 1.24-1.52). It is important to emphasize that the population-level data reported by Wee et al. [26], Lim et al. [7,27], and Chow et al. [8] represent sequential and complementary analyses derived from the same Singapore national registry population. While methodologically robust, these findings reflect a single large population source and should be interpreted as complementary aspects of the same cohort rather than independent geographic validations.

Chow et al. [8] extended follow-up to two years, providing the first population-level quantification of DALY burden attributable to post-acute dengue sequelae, with excess hospitalizations persisting up to 24 months post-infection.

Lim et al. [27] quantified healthcare utilization and cost: an 11.9-fold higher acute inpatient admission rate and a 1.16-fold higher post-acute admission rate, with excess post-acute healthcare costs estimated at USD 687,032 across the cohort over 300 days, representing the first economic-burden estimate for post-acute dengue sequelae.

Neurological Complications

Neurological sequelae are the most frequently represented category (30.6%, n = 19). Principal entities include GBS, dementia, cerebrovascular events, demyelinating disorders, and chronic viral encephalitis.

Kalimuddin et al. [28] followed a prospective cohort of dengue patients in Singapore and found that 18% developed chronic sequelae, including fatigue, impaired concentration, and memory disturbances that persisted at three months. Effiong et al. [29] conducted the first systematic review specifically examining dengue-associated GBS, pooling 56 patients across 36 publications. The mean age was 34.9 years; 57% were male. Symptomatic dengue preceded GBS in 95% of cases, with a median latency of seven days. IVIG was administered in 76.7% of patients and plasmapheresis in 14.3% of patients; 25% of patients required mechanical ventilation. Outcomes were generally favorable: 57% of patients achieved full recovery, 32% achieved partial recovery, and 5.4% died. A high proportion of axonal subtypes was noted, and acute thrombocytopenia was associated with a higher likelihood of requiring ICU care. This systematic review provides the primary evidence source for dengue-associated GBS, complementing the case-report literature summarized in Table 7.

**Table 7:** Summary of studies on neurological complications following dengue infection. GBS: Guillain-Barré syndrome.

Authors	Country/design	Main finding
Hassan et al. [32], 2025	Sudan - case report	Within-series GBS frequency ~9.26%; near-complete recovery at three months.
Effiong et al. [29], 2026	Multi-country - systematic review (n = 56 pts, 36 publications)	Median GBS latency: seven days; 57% full recovery, 32% partial, 5.4% mortality; high axonal subtype; thrombocytopenia predicts ICU need.
Allioni et al. [30], 2025	Brazil - case report + brain metagenomics	First reported case of chronic DENV-1 encephalitis with rapidly progressive dementia; DENV-1 RNA identified in brain tissue by metagenomic sequencing, suggesting a possible cause.
Yoosuf et al. [31], 2025	United Arab Emirates - case report	Dengue with bilateral thalamic diffusion-restriction lesions on MRI; long-term cognitive implications.
Rayamajhi et al. [33], 2024	Nepal - case report	GBS typically two weeks post-dengue; consider in the differential of progressive limb weakness with fever.
Chien et al. [34], 2023	Taiwan - retrospective cohort	Slightly increased risk of non-vascular and total dementia post-dengue.
Bruno et al. [35], 2023	Italy - narrative review	71% higher dementia risk (HR 1.71); markedly elevated in elderly (HR 3.80-12.8).
Chien et al. [36], 2023	Taiwan - retrospective cohort	After 30 days: total stroke risk 6.71 per 1,000 in elderly dengue patients.
Kalimuddin et al. [28], 2022	Singapore - prospective cohort	18% developed chronic sequelae (fatigue, impaired concentration, and memory disturbances) at three months.
Lnu et al. [37], 2022	India - case series	Persistent multifocal dystonia and short-term memory loss post-discharge.
Ganguly et al. [38], 2022	India - case report	Acute hemorrhagic leukoencephalitis and ADEM left chronic lesions.
Basnayake et al. [39], 2021	Sri Lanka - case report	Cerebral ischemia during the acute phase required rehabilitation to approach baseline function at six months.
Badat et al. [40], 2018	England - systematic review	~50% of transverse myelitis cases developed chronic motor sequelae.
Bhushan et al. [41], 2018	India - cross-sectional	14.6% neurological complications; a subset with chronic sequelae (Miller Fisher, ADEM, optic neuritis, etc.).
Higgoda et al. [42], 2018	Sri Lanka - case report	First reported case of multifocal motor neuropathy post-dengue.
Li et al. [43], 2018	Taiwan - retrospective cohort	Significantly higher long-term risk of cerebrovascular events.
Fragoso et al. [3], 2016	Brazil - case report	Acute motor/sensory axonal neuropathy; recovery nine days to one year with early immunoglobulin therapy.
Gonzalez et al. [44], 2015	Colombia - case report	Slow muscle strength recovery beginning at eight weeks post-mechanical ventilation.
Medina-González et al. [45], 2011	Mexico - case report	GBS progressed to cardiorespiratory arrest and persistent vegetative state.

Allioni et al. [30] reported the first case of chronic dengue virus encephalitis with rapidly progressive dementia in which brain biopsy and next-generation sequencing identified DENV-1 RNA in brain tissue, suggesting a possible role in chronic, progressive encephalitis with severe cognitive decline.

Yoosuf et al. [31] described a case of dengue fever with bilateral thalamic diffusion-restricted lesions on MRI, with potential long-term cognitive and sleep-related implications given the anatomical location of the injury.

Ophthalmological Complications

This remains the second most frequently represented category (19.4%, n = 12 of 62) (Table 8). Mbu-Nyamsi et al. [46] reported reduced visual acuity in 15 patients. Several ocular manifestations that initially appear acute evolve into persistent, potentially irreversible sequelae, including permanent reduction in visual acuity, central scotomas, irreversible retinal pigment epithelium changes [47], progressive scleral thinning documented over 18 years [48], and optic atrophy following neuritis or neuroretinitis [49]. The principal entities include macular disorders, optic neuropathy, retinal vasculitis, uveitis (including late-onset uveitis at 2-3 months [47]), and severe structural complications such as corneal ulcers, endophthalmitis, and panophthalmitis [50].

**Table 8:** Summary of studies on ophthalmological complications.

Authors	Country/design	Main finding
Mbu-Nyamsi et al. [46], 2023	France (Reunion) - retrospective	Visual acuity reduction in 15 patients; difficulty reading, night driving, and screen sensitivity.
Abroug et al. [47], 2021	Tunisia - narrative review	Persistent foveal alterations/RPE changes after foveolitis; permanent visual acuity reduction possible.
Vijitha et al. [50], 2021	India - case series	Chronic visual loss with orbital hemorrhage, corneal ulcer, endophthalmitis, or panophthalmitis.
Agarwal and Agarwal [51], 2020	Nepal - case report	Macular vasculitis causes visual acuity reduction via edema/infarction.
Kamoi et al. [48], 2018	Japan - case report	Progressive scleral thinning over 18 years post-scleritis.
Carrillo-Soto [52], 2018	Guatemala - case series	Optic atrophy after neuroretinitis; perifoveal scarring after maculopathy.
Ramos et al. [53], 2015	Spain - case report	Reduced visual acuity in a traveler.
Yip et al. [54], 2012	Singapore - systematic review	Isolated cases with refractory low visual acuity.
Quek et al. [55], 2009	Singapore - case report	Different serotypes may cause posterior uveitis with residual scotomas.
Gupta et al. [56], 2009	India - case report	Late-onset uveitis 2-3 months post-infection.
Sanjay et al. [49], 2008	Singapore - case report	Dengue-associated optic neuropathy can cause permanent visual loss.
Loh et al. [57], 2008	Singapore - case report	Foveal lesion suggesting photoreceptor damage.

Psychiatric Complications

The psychiatric sequelae evidence base includes two comprehensive narrative reviews alongside cohort and observational data (Table 9). D'Souza and D'Silva [58] synthesized 30 studies, representing the largest psychiatric-specific review of dengue sequelae to date, documenting mood disorders, psychosis, anxiety, and body dysmorphic disorder. Implicated mechanisms include cytokine-mediated immune-inflammatory responses, blood-brain barrier disruption, direct viral neurovirulence, and histone-deacetylase-mediated epigenetic dysregulation. Dengue severity, thrombocytopenia, CNS involvement, and DENV-2/DENV-3 serotypes were identified as predictors of psychiatric sequelae.

**Table 9:** Summary of studies on psychiatric complications following dengue infection.

Authors	Country/design	Main finding
Salazar Buenaño et al. [60], 2024	Ecuador - prospective cohort	Higher depression/stress at three months; working memory impairment persisted at 12 months.
Shih et al. [61], 2024	Taiwan - retrospective cohort	Elevated depressive disorder risk; sleep disorders between 3 and 12 months.
D'Souza and D'Silva [58], 2025	India - narrative review (30 studies)	Mood disorders, psychosis, anxiety, and body dysmorphic disorder; DENV-2/3 as predictors; and an HDAC-mediated mechanism proposed.
Lucchese and Stufano [59], 2025	Europe - narrative review	Documents post-infectious neuropsychiatric burden; calls for awareness among European clinicians.
Dinakaran et al. [62], 2022	India - narrative review	Anxiety in 5% at three months; depression persisting 6-24 months; rare catatonia, mania, and psychosis.
Gunathilaka et al. [63], 2018	Sri Lanka - longitudinal descriptive	Higher depression/anxiety/stress vs. controls, documented up to two years.

Lucchese and Stufano [59] called for increased awareness of dengue and chikungunya psychiatric sequelae among mental health professionals in Europe in the context of the northward geographic expansion of arboviral transmission and documented post-infectious neuropsychiatric burden, warranting multidisciplinary follow-up.

Temporal Profile of Chronic Complications

Table 10 summarizes the approximate onset and duration of each category of chronic complication identified in this review, along with the key supporting evidence.

**Table 10:** Summary of onset and duration of chronic complications following dengue infection. Duration ranges are approximate and derived from heterogeneous individual studies with differing follow-up periods and outcome definitions; they do not represent pooled or meta-analyzed estimates. DALY: disability-adjusted life-year.

Complication type	Approx. onset	Approx. duration	Key supporting evidence
Cardiovascular	7-30 days	30-300 days	+46% risk at 31-300 days, population level [7]
Fatigue	2-4 weeks	3-6 months (up to 12 in isolated cases)	[10-14,16]
Heterogeneous	Variable	Fatigue 3-6 months; arthralgia 1-2 months; alopecia up to six months	[15,17-23]
Immunological	Variable	Elevated through ≥300 days; long-term persistence unconfirmed	[7]
Multisystemic	31 days post-infection	Up to 24 months	Two-year DALY burden and excess hospitalizations [8]
Neurological (GBS)	Median seven days from onset	2-6 months (57% full recovery, 32% partial)	Systematic review, n = 56 pooled patients [29]
Neurological (dementia/encephalitis)	Months to years	Indefinite	First confirmed chronic DENV encephalitis [30]
Neuropsychiatric (population cohort)	31 days	Up to 300 days	+29% risk, population level [7]
Ophthalmological	~7 days	Several years or permanent	[46-50]
Psychiatric	~4 weeks	Depression up to six months; cognitive/sleep up to 12 months	Serotype/mechanism predictors [58,59]

Discussion

This scoping review, synthesizing evidence through June 15, 2026, across 62 included studies, finds that neurological complications remain the most frequently represented category in the included literature, consistent with the established neurotropism of DENV and the broad range of immune-mediated mechanisms capable of affecting the central and peripheral nervous systems. The association between dengue and GBS, historically supported mainly by case reports, is now underpinned by a systematic review pooling 56 patients [29], with a median latency of seven days, a high proportion of axonal subtypes, and an incomplete-recovery rate (37.4% of patients experienced partial recovery or death; the remaining ~5.6% were not classified in the pooled analysis) that carries direct implications for post-dengue neurological follow-up in endemic settings.

Taken together, the mechanisms identified across the included studies converge on a multifactorial model of post-dengue chronicity (Table 11). In addition to the cytokine-mediated and autoimmune pathways implicated in the neurological and psychiatric sequelae described above, β-amyloid accumulation and sustained microglial activation following dengue infection have been proposed as potential accelerators of neurodegeneration. Direct central nervous system viral persistence, documented in a single case by metagenomic sequencing that identified DENV RNA in brain tissue [30], and histone deacetylase-mediated epigenetic dysregulation, proposed as a mechanism specific to psychiatric sequelae [58], further extend this pathophysiological model beyond classical neuroinflammatory pathways.

**Table 11:** Proposed pathophysiological mechanisms of chronic post-dengue complications. Several pathways in this table (e.g., cytokine-mediated neuroinflammation, complement activation, and HPA axis dysregulation) represent proposed mechanisms inferred from the clinical or epidemiological associations reported in the cited studies; the cited studies did not directly measure the biological pathway indicated. Direct biological confirmation was available only for CNS viral persistence (metagenomic sequencing) [30] and molecular mimicry in autoimmune encephalomyelitis (autoantibody detection) [24]. This distinction is discussed further in the Discussion section. BBB: blood-brain barrier; CNS: central nervous system; GBS: Guillain-Barré syndrome; HPA: hypothalamic-pituitary-adrenal; HDAC: histone deacetylase; IFN-γ: interferon-gamma; IL: interleukin; NS1: non-structural protein 1; TNF-α: tumor necrosis factor-alpha.

Triggering mechanism	Pathway	Chronic consequences
Direct viral neurotropism (CNS replication)	CNS invasion and glial activation	Encephalitis, seizures, and chronic cognitive impairment [5,6]
Cytokine storm (TNF-α, IL-1, IL-6, IL-8, and IFN-γ)	Neuroinflammation and BBB disruption	Prolonged fatigue, chronic headache, myalgia, and mood disorders [10-14]
Molecular mimicry (autoantibody production)	Demyelination and autoimmunity	GBS, autoimmune encephalomyelitis, and autoimmune neuropathies [24,25,33,44,45]
HPA axis dysregulation	Cortisol imbalance and neuroendocrine alteration	Fatigue, depression, insomnia, and anxiety [60-63]
Complement activation (NS1-mediated and C5b-C9)	Retinal/endothelial apoptosis	Chronic vasculopathy and visual disturbances [46-57]
Autonomic dysregulation	Orthostatic intolerance and heart-rate variability	Cardiac arrhythmia, syncope, and postural tachycardia [7,9,26]
β-amyloid accumulation and chronic microglial activation	Accelerated neurodegeneration	Cognitive decline, Alzheimer's risk, and movement disorders [34,35]
Direct CNS viral persistence	Chronic encephalitis	Rapidly progressive dementia [30]
HDAC-mediated epigenetic dysregulation	Altered gene expression and neuroinflammation	Mood disorders, psychosis, and body dysmorphic disorder [58]

It is important to distinguish, within this proposed multifactorial model, between mechanisms supported by direct biological measurement and those inferred from clinical association alone. Population- and cohort-level studies included in this review, such as the increased risk of dementia [34] and neurological or psychiatric disorders [6] following dengue infection, demonstrate an epidemiological association between dengue and these outcomes but did not directly measure the proposed underlying pathway (e.g., β-amyloid deposition, viral neuroinvasion). By contrast, direct central nervous system viral persistence was confirmed at the molecular level in a single case through brain metagenomic sequencing [30], and immune-mediated mechanisms such as molecular mimicry have direct laboratory support in the autoimmune encephalomyelitis literature [24]. This distinction matters clinically: association-based findings support the rationale for post-dengue surveillance and hypothesis-driven research but should not be interpreted as established causal pathways. Table 11 summarizes the proposed mechanisms and their corresponding chronic consequences.

Population-level evidence from the Singapore registry [7,8] provides quantitative support for the cardiovascular, neuropsychiatric, and autoimmune associations described elsewhere in this review, extending to a two-year horizon and confirming that post-acute sequelae burden is not confined to the early convalescent period; as this evidence originates from a single national registry, it should be regarded as hypothesis-strengthening rather than definitive confirmation until replicated in other populations. At the case level, molecular confirmation of CNS viral persistence [30] and cohort-level dementia associations [34,35] together suggest that neurodegenerative risk after dengue may operate through both direct viral and indirect immune-mediated pathways.

Ophthalmological complications remain the second most represented category in the included literature and continue to highlight that ocular involvement, frequently dismissed as an acute finding, can result in permanent visual impairment; the 18-year follow-up of progressive scleral thinning [48] underscores the need for long-term ophthalmological surveillance. Psychiatric sequelae, previously the least-studied category, have been substantially advanced by D'Souza and D'Silva [58], who identified HDAC-mediated epigenetic dysregulation and DENV-2/DENV-3 serotypes as clinically actionable predictors, suggesting future utility for serotype-informed risk stratification. The wide variability in reported post-dengue fatigue prevalence appears to stem primarily from methodological heterogeneity across studies rather than true differences in incidence, and the consistent female-sex predominance across cohorts warrants dedicated investigation into hormonal-immunological interactions affecting cytokine profiles.

The methodological heterogeneity of the included literature is important when interpreting the apparent distribution of post-dengue complications. Although neurological complications represented the largest proportion of included publications, this finding should not be interpreted as evidence that neurological sequelae are necessarily the most prevalent chronic complications at the population level. The evidence base included prospective and retrospective cohorts as well as case reports and case series, with 56% of studies classified as high quality, 34% as moderate quality, and 10% as low quality according to the JBI assessment. Case-based literature may preferentially capture severe, unusual, or diagnostically distinctive manifestations and therefore may overrepresent certain complication categories. Accordingly, the frequency with which a complication was represented in the literature should be distinguished from its epidemiological frequency among individuals recovering from dengue.

A cross-cutting finding is the absence of a consistent relationship between acute dengue severity and the development of chronic sequelae. Lim et al. [7] reported that the majority of the 55,870 dengue-infected individuals in their cohort had mild initial infections not requiring hospitalization, yet post-acute sequelae were detected across the population. This supports the consideration of structured long-term follow-up for recovered dengue patients, regardless of acute severity, rather than limiting follow-up to patients with severe or hospitalized disease.

Clinical Implications

These findings carry direct implications for post-dengue clinical practice. The considerations below are drawn from heterogeneous, largely observational, and case-level evidence and should be interpreted as areas warranting clinical judgment and further prospective validation, rather than as a standardized surveillance protocol. Structured follow-up may be considered for recovered dengue patients, particularly those with persistent symptoms or clinically relevant risk factors, given the absence of a consistent severity-outcome relationship. Ophthalmological evaluation may be considered within the first two weeks of recovery in patients with persistent or new visual symptoms, given the early onset (~7 days) and potentially permanent nature of ocular complications reported in the case-level literature. Neuropsychiatric screening using validated instruments (e.g., DASS-21, CANTAB cognitive battery) at 3 and 12 months post-infection may be considered for patients with persistent symptoms or clinically relevant risk factors to enable early identification of depressive, anxiety, and working memory deficits; the serotype- and severity-based predictors identified by D'Souza and D'Silva [58] could help target this screening rather than support universal application. Cardiovascular monitoring at 30-300 days is supported by population-level data [7] and should be considered for at-risk patients. The healthcare-cost data from Lim et al. [27] add a further dimension: post-acute dengue sequelae generate measurable excess healthcare expenditure extending through 300 days post-infection, which should inform health-system planning and resource allocation in dengue-endemic countries, particularly where the ratio of recovered patients to available follow-up infrastructure is already strained. The development of evidence-based post-dengue follow-up protocols, particularly within Latin American healthcare systems, remains an urgent and actionable public health priority.

Latin American Context

Latin American countries contributed only 17 of the 62 included studies (27%), a modest share relative to the region's dengue burden. The region of the Americas recorded its most severe dengue epidemic on record in 2024, with more than 12.6 million cases reported [64]. This widens the gap between the region's endemic burden and its representation in the chronic-sequelae literature. The four large Singaporean cohort studies [7,8,26,27], while providing the highest-quality quantitative evidence to date, come from a high-income, island-state setting with healthcare infrastructure, serotype distribution, and population characteristics that differ substantially from those of Ecuador, Brazil, Colombia, or Peru. Prospective cohort studies based in Latin American settings remain urgently needed. The predominance of Asian cohort data underscores a critical evidence gap: regional differences in dominant circulating serotypes (e.g., DENV-1 and DENV-2 dynamics), high rates of secondary or tertiary infections, viral co-circulation (such as Zika, Chikungunya, and Oropouche), and distinct healthcare infrastructure across Latin America may substantially alter both the clinical phenotype and long-term burden of chronic sequelae compared to Asian populations.

Limitations

The predominance of case reports and case series among the included studies (approximately one-third) limits causal inference and prevalence estimation. This case-based predominance may also introduce publication bias toward severe, atypical, or diagnostically striking presentations (e.g., fatal encephalitis, extensive GBS), while milder or non-specific chronic manifestations such as low-grade fatigue may be underrepresented in the published literature, potentially skewing the apparent severity spectrum of post-dengue chronic complications. It should also be noted that the four largest cohort studies [7,8,26,27] appear to draw on the same underlying Singapore national registry population (2017-2023); this evidence should therefore be interpreted as originating from a single population-level data source rather than as fully independent confirmatory studies. Geographic heterogeneity remains pronounced, with Latin American settings underrepresented despite bearing a disproportionate share of the global disease burden, which constrains the generalizability of the current evidence base to that region. Definitional heterogeneity in what constitutes a chronic complication and variation in follow-up durations continue to complicate cross-study comparisons; we applied a consistent four-week threshold throughout this review (see Introduction) to standardize synthesis across studies with differing original definitions. Additionally, while comprehensive search strategies across multiple databases were deployed using institutional and open-access retrieval, access restrictions to specific paywalled journals not indexed in our subscriptions may have inadvertently omitted a minority of eligible studies, presenting a minor potential selection bias. Furthermore, language filters restricted to English and Spanish may have excluded relevant literature published in other languages, particularly Portuguese-language studies from Brazil, a country with a substantial dengue burden and already represented in this review; this may have led to underrepresentation of Brazilian chronic-sequelae literature not indexed or published in English/Spanish.

EMBASE, accessed via institutional access, contributed 12 raw records, most of which overlapped with studies identified through PubMed. The relatively small number of records retrieved from EMBASE may reflect substantial overlap in indexing across databases and the eligibility criteria applied in this review. An expected lag between publication and bibliographic indexing means that some eligible studies from the final months of the search period may not have been captured. Finally, heterogeneity in study designs, follow-up durations, and outcome definitions across all 62 included studies precludes quantitative synthesis or pooled prevalence estimation; reported prevalence ranges should be interpreted with appropriate caution.

Retrospective OSF registration (osf.io/7dvuj, registered May 30, 2026) is acknowledged as a methodological limitation because database searches had already started before registration. However, the registration provides a transparent record of the review protocol and prespecified methodological framework. The eight complication categories were defined during the planning and piloting stages based on established clinical and pathophysiological domains and were not expanded in response to findings identified during the search. Therefore, no post-hoc complication categories were added.

Similarly, the comparatively low yields from Scopus (n = 9), Cochrane Library (n = 1), and LILACS (n = 4) may partly reflect overlap with records identified through PubMed (n = 173). This distribution (PubMed 173/222, 78% of raw records) reflects the fact that PubMed comprehensively indexes MEDLINE-listed journals covering dengue research; the low yield from other databases should not be interpreted as an absence of relevant literature in those sources, but rather as substantial indexing overlap with PubMed. The low yield from the Cochrane Library may also be related to the predominance of observational studies, case reports, and case series in this review, as this database primarily indexes systematic reviews and evidence syntheses relevant to clinical interventions. The relatively low yield from LILACS should be interpreted cautiously and does not necessarily indicate limited Latin American research on chronic or post-dengue complications.

## Conclusions

Taken together, these findings demonstrate that dengue infection is associated with a broad and heterogeneous spectrum of persistent and chronic complications affecting multiple organ systems, with reported sequelae extending up to two years post-infection. The maturation of dengue-associated GBS evidence, from isolated case reports to a pooled systematic review, illustrates a broader pattern in this field: as case-level observations accumulate, they increasingly support mechanistic and prognostic claims that were previously speculative. The substantial proportion of patients with incomplete recovery in that pooled analysis reinforces the case for incorporating neurological follow-up into standard post-dengue care in endemic settings, rather than treating GBS as an exceptional or unpredictable event.

The absence of a consistent relationship between acute severity and chronic outcomes, including findings from a cohort of nearly 56,000 adults, suggests that persistent complications may occur across the spectrum of acute dengue severity. These findings may have implications for post-dengue follow-up, particularly among patients with persistent or recurrent symptoms. The available evidence does not establish a uniform surveillance schedule for all dengue survivors; rather, it supports consideration of individualized follow-up according to persistent manifestations, severity of the acute illness, relevant comorbidities, and other clinical risk factors. Patients with persistent neurological, psychiatric, visual, cardiovascular, or musculoskeletal symptoms may warrant targeted evaluation and specialist referral as clinically indicated. Prospective longitudinal studies are needed to determine whether standardized post-dengue surveillance strategies improve the detection and management of persistent complications. Latin American populations remain underrepresented in the available literature, which may limit the generalizability of current findings to settings where dengue represents a substantial public health burden. As the strongest population-level evidence in this review originates from a single national registry, independent confirmation in other populations remains a priority for future research.

## Appendices

**Table 12:** Complete search strategies by database. No exclusion filter was applied at the search-string level for "acute"; acute-only studies were instead excluded manually during full-text screening (see Eligibility Criteria) to avoid inadvertently excluding eligible chronic-sequelae studies that also mention acute-phase findings.

Database	Records	Search string/notes	Filters applied
PubMed	173	("dengue"[MeSH] OR "dengue fever"[tiab] OR "DENV"[tiab]) AND ("chronic"[tiab] OR "long term"[tiab] OR "sequelae"[tiab] OR "post-infection"[tiab] OR "complications"[tiab]) NOT ("acute"[tiab] AND NOT "chronic"[tiab])	January 2005 to June 15, 2026; English/Spanish; observational, case report, and systematic/narrative review; institutional/OA access.
Scopus	9	TITLE-ABS-KEY("dengue" AND ("chronic complications" OR "long-term sequelae" OR "post-dengue" OR "post-infection" OR "sequelae"))	2005-2026; English/Spanish; article, review
Google Scholar	21	"dengue" AND ("chronic complications" OR "sequelae" OR "post-dengue syndrome" OR "long term" OR "post-infection")	2005-2026; first 20 pages; dedup via Rayyan
EMBASE	12	TITLE-ABS-KEY('dengue' AND ("chronic complications" OR "long-term sequelae" OR "post-dengue" OR "sequelae"))	2005-2026; English/Spanish; institutional access
Cochrane Library	1	"dengue" AND ("sequelae" OR "chronic" OR "long-term complications")	Title/abstract/keywords; 2005-2026
LILACS	4	"dengue" AND ("secuelas crónicas" OR "complicaciones crónicas" OR "post-dengue" OR "sequelae")	Virtual Health Library (VHL)/Biblioteca Virtual en Salud (BVS) interface; 2005-2026; English/Spanish
Gray literature	2	Manual search, tropical medicine societies, and university repositories	Brazil, Colombia, Ecuador, Mexico, and Peru; 2005-2026
Total	222	-	-
